# Assessment of Kynurenine Pathway Enzyme Activity in Ocular Diseases: Associations with Cataract, Diabetes, Glaucoma, and Pseudoexfoliation Syndrome

**DOI:** 10.3390/jcm14134529

**Published:** 2025-06-26

**Authors:** Arturs Zemitis, Juris Vanags, Kristaps Klavins, Guna Laganovska

**Affiliations:** 1 Department of Ophthalmology, Riga Stradins University, LV 1007 Riga, Latvia; 2 Clinic of Ophthalmology, Pauls Stradins Clinical University Hospital, LV 1002 Riga, Latvia; 3 Institute of Biomaterials and Bioengineering, Faculty of Natural Sciences and Technology, Riga Technical University, LV 1048 Riga, Latvia

**Keywords:** kynurenine pathway, cataract, glaucoma, diabetes, kynurenine aminotransferase, kynurenine monooxygenase, 3-Hydroxykynurenine, kynurenic acid, oxidative stress, ocular metabolism

## Abstract

**Purpose:** To investigate the role of the kynurenine pathway (KP) in ocular diseases by evaluating the activity of key enzymes—kynurenine aminotransferase (KAT) and kynurenine monooxygenase (KMO)—and the 3-hydroxykynurenine to kynurenic acid (3-HK/KYNA) ratio in relation to cataract severity, diabetes, glaucoma, and pseudoexfoliation syndrome (PEXS). **Methods:** Tryptophan metabolite levels were measured in patients undergoing cataract surgery and stratified by SPONCS grading and comorbid conditions. KAT and KMO activities were estimated using metabolite ratios (KYNA/KYN and 3-HK/KYN, respectively). Statistical analyses included Kruskal–Wallis tests with post hoc comparisons and Mann–Whitney U tests. **Results:** KAT activity declined significantly with increasing SPONCS grade (*p* = 0.014), suggesting a progressive loss of KYNA production and antioxidative capacity in advanced cataracts. Diabetic patients exhibited higher KMO activity (*p* = 0.039) and elevated 3-HK/KYNA ratios (*p* = 0.013), indicating a metabolic shift toward oxidative stress and neurotoxicity. Similarly, glaucoma patients had significantly increased KMO activity (*p* = 0.032), consistent with enhanced 3-HK-mediated retinal ganglion cell damage. In contrast, PEXS showed no significant alterations in KP markers. **Conclusions:** The kynurenine pathway is differentially modulated in ocular diseases. A decline in KAT activity correlates with cataract severity, while upregulation of KMO is prominent in diabetes and glaucoma, revealing disease-specific metabolic dysregulation. Targeting KMO to reduce toxic metabolite accumulation or enhancing KYNA synthesis may offer novel therapeutic avenues. These findings also support the potential of KP metabolites as biomarkers for disease monitoring and progression.

## 1. Introduction

The kynurenine pathway (KP) is the principal route of tryptophan catabolism, generating metabolites that serve as UV filters in the lens, modulators of neurotransmission in the retina, and mediators of redox balance throughout the eye [1]. This pathway generates various metabolites, such as kynurenine (KYN), kynurenic acid (KYNA), and 3-hydroxykynurenine (3-HK), which have been implicated in both protective and harmful roles in various physiological and pathological processes [2]. Kynurenines such as 3-HK absorb ultraviolet light but can undergo oxidative transformation to form protein-bound adducts, contributing to lens opacification and cataractogenesis [3,4]. Elevated levels of KP metabolites, such as KYN and 3-HK, have been observed in the lenses of patients with cataracts. These metabolites can modify lens proteins, leading to oxidative stress and cross-linking of α-crystallins, which are critical for maintaining lens transparency [5].

Kynurenine aminotransferases (KATs) direct flux toward KYNA, a broad-spectrum antagonist of excitatory amino acid receptors with documented neuroprotective and antioxidant effects [6,7]. In retinal models, elevated KYNA levels—whether via KAT activity or Kynurenine 3-monooxygenase (KMO) inhibition—mitigate ischemia/reperfusion injury and protect retinal ganglion cells from excitotoxic death [8]. KMO catalyzes the conversion of KYN to 3-HK, a neurotoxic precursor of quinolinic acid and reactive oxygen species [9]. Dysregulated KMO activity and subsequent 3-HK accumulation have been implicated in both diabetic cataract formation and glaucomatous retinal damage, linking metabolic flux through this branch to ocular oxidative stress [10].

Nuclear cataract severity is objectively quantified by the Simplified Pre-Operative Nuclear Classification Score (SPONCS), a reproducible grading system strongly correlated with lens density and intraoperative outcomes [11]. Despite growing interest in KP metabolites as biomarkers, no study has yet mapped KAT and KMO activities across SPONCS grades to elucidate how flux through each branch relates to cataract severity. In systemic disease, diabetes mellitus hyperactivates indoleamine 2,3-dioxygenase (IDO) and downstream KP enzymes, driving KMO flux and oxidative stress in both lens and retinal tissues [12]. Recent work demonstrated progressive increases in aqueous humor IDO activity correlating with cataract severity (SPONCS 2 vs. SPONCS 5, *p* = 0.013), suggesting kynurenine pathway activation contributes to oxidative stress in lens epithelial cells during cataractogenesis [13]. In diabetic patients, the accumulation of KYN and its metabolites in the aqueous humor and lenses is significantly higher compared to non-diabetic individuals. This accumulation is associated with oxidative damage and advanced glycation end-product (AGE) formation, which accelerates cataract progression [14]. Glaucoma is characterized by progressive retinal ganglion cell loss and optic nerve degeneration [15]. Its pathogenesis involves oxidative damage and excitotoxicity to retinal ganglion cells, processes that may be exacerbated by an imbalance between KAT and KMO-mediated metabolites [16]. Studies have shown that the levels of KYN and its derivatives, such as KYNA, are significantly altered in the aqueous humor of glaucoma patients. These changes are associated with mitochondrial dysfunction and oxidative stress, which contribute to retinal ganglion cell (RGC) death [17]. KP imbalance exacerbates retinal neurodegeneration: preclinical studies show that optic nerve injury or NMDA-induced RGC death triggers downregulation of KAT isoforms and transient KMO upregulation, leading to elevated 3-HK and depleted KYNA [1]. In a murine model of hereditary glaucoma, reduced levels of tryptophan and KP metabolites, such as KYNA and KYN, were observed in the retina. This suggests that imbalances in the KP may play a role in the progression of glaucoma [18]. Conversely, pseudoexfoliation syndrome (PEXS) is characterized by extracellular matrix abnormalities and oxidative stress, but its relationship to KP enzyme activity has not been clearly defined [19]. While PEXS has long been associated with oxidative stress, recent metabolomic evidence reveals a potential link to ferroptosis [20].

Our study aims to evaluate the relationship between KP enzyme activities and key ocular phenotypes by correlating KAT and KMO activities measured indirectly via KYNA/KYN and 3-HK/KYN ratios with nuclear cataract severity as graded by SPONCS and determining how these activities differ in patients with diabetes, glaucoma, and PEXS. We also assess the 3-HK/KYNA ratio as an index of the balance between the neurotoxic (KMO-driven) and neuroprotective (KAT-driven) branches of the pathway. By linking enzymatic flux through each branch of the pathway to clinical measures of disease, we seek to clarify the contribution of KP dysregulation to ocular pathology and identify potential targets for therapeutic intervention.

## 2. Materials and Methods

### 2.1. Study Group

This cross-sectional investigation examined aqueous humor specimens obtained from 101 individuals undergoing cataract surgery at Pauls Stradiņš Clinical University Hospital. The cohort comprised 32 male and 69 female participants, with a mean age of 74.7 years (±9.06 SD) and an age range of 50–94 years. Cataract severity was evaluated using the SPONCS classification system, which hierarchically categorizes nuclear opalescence and coloration across eight incremental stages (SPONCS 1 to 5, with intermediate “+” designations). Patients were categorized by their SPONCS classification levels. The study included 34 patients (33.7%) with SPONCS 2/2+, 33 patients (32.7%) with SPONCS 3/3+, 12 patients (11.9%) with SPONCS 4/4+, and 22 patients (21.8%) with SPONCS 5. Patients with the mildest classification (SPONCS 1) were not included in the study.

The study included participants with age-related macular degeneration. However, since baseline AMD status was not systematically recorded prior to surgery, this represents an important limitation in our data collection that may affect the interpretation of results. Although AMD patients were present in the cohort, the absence of standardized phenotyping (e.g., AREDS classification, OCT/angiography) precluded subgroup analysis. We posit that serum KP metabolites—reflecting systemic inflammation—may better correlate with AMD progression than aqueous humor, given its posterior segment locus. Future studies should prioritize serum KP profiling in dedicated AMD cohorts. The distribution of ocular comorbidities and their relationships within the study cohort are detailed in Table 1, Table 2, Table 3 and Table 4.

Prior to surgery, aqueous humor samples were collected via paracentesis using a 27-gauge needle. Depending on availability, between 50 and 120 µL of fluid was carefully aspirated and transferred into sterile Eppendorf tubes. The samples were initially frozen at −18 °C on-site and transported the same day in insulated iceboxes to the Faculty of Materials Science and Applied Chemistry at Riga Technical University. There, they underwent further analysis and were stored long-term at −80 °C to preserve sample integrity.

Ethical oversight was provided by the Rīga Stradiņš University Medical Ethics Committee (Approval No. 2-PEK-4/307/2023, dated 21 March 2023), with operational approval granted by Pauls Stradiņš Clinical University Hospital. All participants provided written informed consent, and the study was conducted in full compliance with the ethical principles outlined in the Declaration of Helsinki.

### 2.2. LC-MS-Based Metabolite Analysis

Aqueous humor metabolites were isolated via methanol-based extraction. Briefly, 10 µL of each sample was aliquoted into Eppendorf tubes and combined with 80 µL methanol and 10 µL isotope-labeled internal standards. The mixture was vortex-mixed for 15 s, followed by centrifugation (10,000 RPM, 10 min) to pellet insoluble debris. The clarified supernatant was collected and transferred to HPLC vials for downstream analysis.

Quantitative metabolite profiling was performed using hydrophilic interaction liquid chromatography (HILIC) coupled to a high-resolution Orbitrap Exploris 120 mass spectrometer (Thermo Fisher Scientific, Waltham, MA, USA). Chromatographic separation was achieved on an ACQUITY UPLC BEH Amide column (2.1 × 100 mm, 1.7 μm; Waters, Milford, MA, USA) maintained at 40 °C. The mobile phase comprised (A) 0.15% formic acid and 10 mM ammonium formate in water and (B) 0.15% formic acid and 10 mM ammonium formate in 85% acetonitrile. A stepwise gradient was applied as follows: initial conditions at 100% B (0–6.0 min), followed by a rapid reduction to 94.1% B (6.0–6.1 min), a gradual decline to 82.4% B (6.1–10.0 min), and a final decrease to 70.6% B (10.0–12.0 min). The column was re-equilibrated at 100% B for 6 min, yielding a total run time of 18 min. The flow rate was fixed at 0.4 mL/min, with an injection volume of 2 μL.

Electrospray ionization was conducted in both positive and negative modes, with spray voltages set to 3.5 kV (positive) and 2.5 kV (negative). Full-scan MS data were acquired across a mass range of 50–600 *m*/*z* at a resolution of 60,000. Operational parameters included a capillary temperature of 350 °C, auxiliary gas flow rate of 12 arbitrary units, nebulizer gas flow rate of 50 arbitrary units, and heater temperature of 400 °C. Quantitation relied on seven-point calibration curves with internal standardization, processed using TraceFinder 5.1.1 software (Thermo Fisher Scientific).

Metabolites were unequivocally confirmed at identification level A [21] by matching retention times (RT) and accurate *m*/*z* values (<5 ppm error) to authenticated reference standards analyzed under identical conditions.

### 2.3. Enzyme Identification

KAT activity was estimated indirectly by calculating the ratio of KYNA to KYN. The KYNA/KYN ratio is widely used as a surrogate for net KAT activity in biological samples [22,23]. While this ratio provides a useful index of net transamination activity directed toward KYNA formation, it does not fully represent the intrinsic catalytic properties of the KAT enzymes. Specifically, the KYNA/KYN ratio is influenced by factors such as the availability of the cofactor pyridoxal 5′-phosphate, the presence and concentration of the necessary 2-oxoacid cosubstrate, and the relative activities of competing metabolic pathways (e.g., the metabolism of 3-HK to xanthurenic acid) [24,25]. Moreover, it does not provide kinetic parameters such as V_max_ and K_m_, which are critical for a comprehensive characterization of enzyme function. Therefore, while changes in this ratio reflect alterations in net KYNA production, they do not completely capture the full scope of KAT enzymatic activity.

KMO activity was estimated indirectly by calculating the ratio of 3-HK to KYN. The 3-HK/KYN ratio serves as a practical index of KMO-mediated hydroxylation of KYN to 3-HK [26]. While this ratio serves as a practical indicator of net KMO activity, it does not fully represent the intrinsic kinetic properties of the enzyme, which can be influenced by cofactor availability, competing metabolic pathways, and the downstream metabolism of 3-HK. KMO relies on NADPH and FAD; fluctuations in cellular redox status can decouple the ratio from true enzyme capacity [27]. Thus, although useful for relative comparisons under our experimental conditions, the 3-HK/KYN ratio represents an indirect measure of KMO activity.

In our study, we also calculated the ratio of 3-HK to KYNA as an index of the balance between the neurotoxic and neuroprotective arms of the KP. Reflects the balance between neurotoxic (KMO) and neuroprotective (KAT) branches [28]. Although this ratio serves as a useful biomarker, reflecting the relative flux through KMO compared to KAT-mediated reactions, it should be interpreted with caution. The 3-HK/KYNA ratio is affected by the availability of substrates, cofactors, and the activity of competing metabolic routes, and therefore, it provides an indirect measure of enzymatic control rather than a direct kinetic assessment of KMO or KAT activity.

Using metabolite ratios such as KYNA/KYN for KAT activity and 3-HK/KYN for KMO activity is a common indirect approach that offers practical insights into pathway flux but does not substitute for direct kinetic characterization. These ratios are influenced by cofactor (e.g., pyridoxal-5′-phosphate, NADPH/FAD) availability, cosubstrate concentrations, and competing metabolic branches (e.g., xanthurenic or quinolinic acid formation).

### 2.4. Statistics

Normality of the data distribution was assessed using the Shapiro–Wilk test. For data that did not conform to a normal distribution, non-parametric statistical methods were employed. Specifically, the Mann–Whitney U test was used to compare differences between two independent groups. For comparisons involving more than two groups, a non-parametric one-way ANOVA (Kruskal–Wallis test) was applied. All statistical analyses were performed using the Jamovi 2.6.25. software platform. Data tables were prepared in Microsoft Excel. Notably, metabolite concentrations obtained via MS-LC were analyzed as raw values without normalization.

## 3. Results

In our study, KAT activity was different between groups [X^2^(3) = 10.63, *p* = 0.014, *ε*^2^ = 0.1063]. Specifically, the median KAT activities were as follows: SPONCS 2/2+—1.598 (IQR: 0.974–2.47), SPONCS 3/3+—1.273 (IQR: 0.776–1.93), SPONCS 4/4+—1.192 (IQR: 0.726–1.62), and SPONCS 5—0.738 (IQR: 0.231–1.50). Post hoc analysis revealed that the largest difference was between SPONCS 2 and SPONCS 5 (*p* = 0.017). In contrast, neither KMO activity nor the 3-HK/KYNA ratio differed among the cataract groups. The observed decrease in KAT activity from SPONCS 2 to SPONCS 5 reveals a shift in the KP away from the production of KYNA, a neuroprotective metabolite. This shift could contribute to increased oxidative stress and neurotoxicity, potentially exacerbating cataract severity.

When comparing patients with and without diabetes, we found that KMO activity was increased more in diabetic patients (median = 0.765, IQR = 0.498–1.095) than in non-diabetic patients (median = 0.545, IQR = 0.364–0.802); this was supported by a Mann–Whitney U test (*U* = 820, *p* = 0.039, *r* = 0.257). Moreover, the 3-HK/KYNA ratio was elevated in diabetic patients (median = 0.668, IQR = 0.306–1.215) compared to those without diabetes (median = 0.401, IQR = 0.401–0.717; *U* = 764, *p* = 0.013, *r* = 0.308). KAT activity, however, did not show significant differences in relation to diabetes status. Elevated KMO activity and a higher 3-HK/KYNA ratio in diabetic patients indicate a metabolic shift toward the production of 3-HK, a metabolite associated with oxidative stress and neurotoxicity. This alteration may contribute to the development of diabetic cataracts and other ocular complications associated with diabetes.

For patients with glaucoma, KMO activity was elevated (median = 0.819, IQR = 0.601–1.085) compared to non-glaucoma patients (median = 0.526, IQR = 0.368–0.824; U = 558, *p* = 0.032, *r* = 0.3111), while no statistically significant differences were observed for KAT activity or the 3-HK/KYNA ratio between the glaucoma groups. The increased KMO activity observed in glaucoma patients suggests enhanced production of neurotoxic metabolites like 3-HK, which could contribute to RGC damage and optic nerve degeneration characteristic of glaucoma.

Lastly, in the group with PEXS, none of the measured values (KAT activity, KMO activity, or the 3-HK/KYNA ratio) reached statistical significance. The lack of changes in KAT and KMO activities or the 3-HK/KYNA ratio in PEXS patients implies that the KP may not be directly involved in the pathogenesis of PEXS. This aligns with studies indicating that PEX is associated with other mechanisms, such as oxidative stress and extracellular matrix abnormalities.

Overall, while the KYNA/KYN ratio (for estimating KAT activity) and the 3-HK/KYNA ratio (for assessing the balance between the neurotoxic and neuroprotective branches of the pathway) provide valuable indirect measures, our findings indicate that alterations are mainly observed in KMO activity in specific subgroups (i.e., diabetes and glaucoma) and in KAT activity across SPONCS severity levels. These indices should be interpreted within the context of the limitations inherent to indirect biochemical assays.

## 4. Discussion

### 4.1. Cataract

Progressive diminution of KAT activity from SPONCS grade 2 through grade 5 cataracts implies a stage-dependent reprogramming of lens tryptophan metabolism that parallels disease progression. In the earliest stage (SPONCS 2), elevated KAT activity may represent an intrinsic antioxidative mechanism [29]: by transaminating KYN to KYNA, KAT enzymes reduce ROS accumulation and slow protein aggregation. As cataract severity advances, the observed, stepwise attenuation of KAT activity likely reflects cumulative oxidative insult and progressive depletion of essential cofactors—most notably pyridoxal phosphate—or direct oxidative inactivation of the enzyme complex. By SPONCS 5, this decline in KAT function not only impairs KYNA-mediated photoprotection but also shunts KYN metabolism toward neurotoxic intermediates, such as 3-HK and quinolinic acid, which have been implicated in crystallin cross-linking and exacerbation of lens opacity in cataracts [30]. Moreover, reduced KAT activity may exacerbate NAD^+^ depletion [31,32], undermining lens cellular repair processes and redox equilibrium.

Despite the progressive decrease in KAT activity, KMO activity and the systemic 3-HK/KYNA ratio remain ostensibly unaltered across SPONCS grades, suggesting either stringent regulatory control of these enzymatic branches or enhanced catabolism of their metabolites. This differential vulnerability underscores the pivotal role of KAT in maintaining lens transparency, whereas broader modulation of the KP may necessitate complementary interventions targeting multiple enzymatic nodes.

Given the modest effect size observed (*ε*^2^ = 0.1063), it is probable that additional variables—such as genetic polymorphisms, ultraviolet light exposure, or metabolic comorbidities—contribute to interindividual disparities in KAT activity. Accordingly, future investigations should incorporate targeted metabolomic analyses of aqueous humor or ex vivo lens tissue to substantiate these findings. From a translational perspective, early-stage (SPONCS 2/2+) lenses may benefit from pharmacologic strategies aimed at preserving KAT cofactor availability (for example, vitamin B_6_ supplementation), whereas advanced-stage cataracts might require adjunctive therapies to scavenge 3-HK-derived radicals or replenish NAD^+^ pools, thereby restoring redox homeostasis and decelerating opacification.

### 4.2. Diabetes

Our study reveals significant dysregulation of the KP marked by elevated KMO activity and an increased 3-HK/KYNA ratio. These alterations suggest a diabetes-induced metabolic shift favoring 3-HK production. This shift may enhance local oxidative stress within the eye, potentially contributing to the pathogenesis of diabetic ocular complications [33].

Chronic hyperglycemia is known to induce oxidative stress and inflammation—factors that can upregulate KMO expression via pathways involving NF-κB [34] or HIF-1α [35]. Increased KMO activity accelerates the conversion of KYN to 3-HK and downstream toxic metabolites such as quinolinic acid. This could lead to direct damage of ocular structures through mechanisms including protein glycation—whereby 3-HK reacts with lens crystallins to form AGEs [36] and mitochondrial dysfunction via the generation of ROS, both of which contribute to lens opacification and retinal injury.

The elevated 3-HK/KYNA ratio observed in diabetic patients highlights an imbalance between the neurotoxic and neuroprotective branches of the KP [37]. While KAT activity appears preserved, KYNA levels may be reduced due to increased consumption as an antioxidant or degradation under hyperglycemic oxidative stress. This selective disruption of the KMO-dependent arm of the KP is consistent with prior studies indicating tissue-specific metabolic alterations in diabetic conditions [38].

These alterations have functional consequences. In cataracts, 3-HK-driven AGE formation likely accelerates lens clouding, while in diabetic retinopathy, increased ROS and pro-inflammatory signaling linked to 3-HK may compromise the blood–retinal barrier and promote diabetic retinopathy via VEGF activation [39]. In patients with comorbid glaucoma, heightened KMO activity may synergize with glaucomatous damage by exacerbating RGC loss through oxidative and excitotoxic mechanisms [40].

These findings underscore the need for further mechanistic and clinical investigations. Future research should focus on quantifying downstream metabolites (e.g., quinolinic acid, NAD^+^), assessing KMO transcriptional regulation in diabetic ocular tissues, and evaluating the prognostic value of KP biomarkers in tracking disease progression. Additionally, therapeutic strategies targeting this pathway—such as KMO inhibitors, KYNA analogs, or antioxidant adjuvants—warrant exploration for their potential to mitigate diabetes-associated ocular disease.

### 4.3. Glaucoma

The marked elevation of KMO activity in glaucoma patients (*p* = 0.032) points to a disease-associated shift in tryptophan metabolism toward the production of neurotoxic metabolites. By catalyzing the conversion of KYN into 3-HK. Elevated activity of KMO may exacerbate glutamate-induced excitotoxicity and promote the generation of ROS, thereby contributing to RGCs degeneration, accelerating optic nerve injury beyond what intraocular pressure alone would predict [41]. The observed increase in KMO activity and the elevated 3-HK/KYNA ratio in patients with glaucoma indicate a potential involvement of KP dysregulation in promoting fibrotic remodeling of the trabecular meshwork. Notably, 3-HK is a pro-oxidant metabolite known to stimulate the release of TGF-β1 and enhance collagen deposition in trabecular meshwork cells, processes that may contribute to ECM stiffening [42]. These findings implicate the KP in both neurodegenerative and fibrotic mechanisms underlying glaucoma pathophysiology and highlight its components as promising therapeutic targets for modulating disease progression.

Despite the rise in KMO activity, the 3-HK/KYNA ratio remains stable, implying compensatory regulation that could mask imbalances within the retina or optic nerve head. Small, cross-sectional patient cohorts raise concerns about statistical power and the potential for false positives. Many glaucoma patients are on intraocular pressure-lowering medications that can independently influence metabolic pathways, introducing confounding factors that complicate causal inference.

Pro-inflammatory cytokines [43] (TNF-α, IL-6, IFN-γ) and chronic hypoxia [44] from reduced ocular perfusion remain plausible upstream drivers of KMO upregulation. Similarly, although small-molecule KMO inhibitors (such as CHDI-340246) have shown promise in Huntington’s models [45], their ocular bioavailability, safety profile, and efficacy in rodent models of ocular hypertension remain untested.

To bridge these gaps, future work should (1) quantify KMO activity and downstream metabolites directly in ocular fluids or post-mortem retina, (2) expand and diversify patient cohorts with rigorous adjustment for multiple comparisons, (3) employ longitudinal designs linking baseline KMO levels to rates of visual field loss and RNFL thinning, and (4) evaluate KMO inhibitors—alone and in combination with NMDA antagonists (e.g., memantine) and antioxidants (e.g., N-acetylcysteine)—in glaucoma-relevant animal models. Such studies will be essential to determine whether targeting the KP can deliver meaningful neuroprotection in glaucoma. Modulating the KP to reduce neurotoxic metabolites and enhance neuroprotective ones, such as KYNA, may provide a therapeutic strategy for glaucoma. Additionally, targeting enzymes like IDO and KMO could help restore balance to the KP and mitigate oxidative stress [1,46].

### 4.4. Pseudoexfoliation Syndrome

PEXS exhibited no significant changes in KP metrics, including KAT activity, KMO activity, and the 3-HK/KYNA ratio. This preservation of pathway balance reveals that KYN metabolism is not a central driver of PEXS pathogenesis. Instead, PEXS appears to be mediated by non-metabolic mechanisms, primarily involving structural abnormalities in the extracellular matrix (ECM) and localized oxidative stress [47].

PEXS is characterized by the deposition of fibrillar material in ocular tissues, a process closely linked to genetic variants in LOXL1 [48] and dysregulation of TGF-β signaling [49].

The maintained 3-HK/KYNA ratio further supports the notion that compensatory mechanisms may preserve pathway homeostasis in PEXS. Balanced expression of both KAT and KMO, or reduced systemic inflammation compared to other ocular diseases, may prevent the skewing of tryptophan metabolism toward either oxidative or neuroprotective arms.

## 5. Conclusions

Our study demonstrates that specific kynurenine pathway (KP) disruptions drive distinct ocular pathologies: cataract progression shows a significant decline in KAT activity (KYNA/KYN ratio decreasing with SPONCS grade, *p* = 0.014), while glaucoma and diabetes exhibit elevated KMO activity (increased 3-HK/KYN ratios, *p* = 0.032 and *p* = 0.039, respectively) and neurotoxic 3-HK/KYNA imbalance (*p* = 0.013), with comorbid cases showing amplified effects. In contrast, PEXS displays no KP alterations, confirming the pathway’s disease-specific involvement. Future studies should explore correlations between serum and aqueous humor KP metabolite levels to establish minimally invasive biomarkers for ocular pathology monitoring and therapeutic response assessment.

These insights open new avenues for therapeutic intervention. Modulating key enzymes—such as inhibiting KMO to reduce 3-HK toxicity or augmenting KYNA production to enhance neuroprotection—could represent a promising strategy to mitigate vision-threatening complications in at-risk populations. Furthermore, KYN metabolites may serve as accessible biomarkers in the aqueous humor for early diagnosis, disease monitoring, and therapeutic response assessment. Future research should aim to validate these targets in longitudinal clinical cohorts and explore the utility of kynurenine-modulating agents in preclinical and clinical settings.

## Figures and Tables

**Table 1:** Comparison of age, sex, and ocular comorbidities across SPONCS severity groups.

	SPONCS 2/2+ (n = 34)	SPONCS 3/3+ (n = 33)	SPONCS 4/4+ (n = 12)	SPONCS 5 (n = 22)	*p*-Value
Age, mean (SD)	72.1 ± 9.95	78.2 ± 6.39	77.5 ± 8.36	71.0 ± 9.76	**0.021**
Gender					0.205
Male	10 (9.9%)	8 (7.9%)	3 (3.0%)	11 (10.9%)	
Female	24 (14.1%)	25 (24.8%)	9 (8.9%)	11 (10.9%)	
Glaucoma					0.256
Present	6 (5.9%)	10 (9.9%)	2 (14.1%	2 (2.0%)	
Absent	28 (27.7%)	23 (22.8%)	10 (9.9%)	20 (19.8%)	
Pseudoexfoliation syndrome					**0.011**
Present	9 (8.9%)	22 (21.8%)	5 (10.9%)	11 (10.9%)	
Absent	25 (24.8%)	11 (10.9%)	7 (6.9%)	11 (10.9%)	
Diabetes					0.796
Present	9 (8.9%)	11 (10.9%)	5 (5.0%)	7 (6.9%)	
Absent	25 (24.8%)	22 (21.8%)	7 (6.9%)	15 (14.9%)	

Age distributions were compared using one-way ANOVA, while categorical variables were analyzed with chi-square tests. Bold *p*-values denote statistically significant differences (*p* < 0.05).

**Table 2:** Association between glaucoma status and patient demographics, cataract severity, diabetes, and pseudoexfoliation syndrome.

	Glaucoma (n = 20)	No Glaucoma (n = 81)	*p*-Value
Age, mean (SD)	74.2 ± 7.80	74.9 ± 9.38	0.771
Gender			0.857
Male	6 (5.9%)	26 (13.9%)	
Female	14 (13.9%)	55 (54.5%)	
Cataract severity			0.256
SPONCS 2/2+	6 (5.9%)	28 (27.7%)	
SPONCS 3/3+	10 (9.9%)	23 (22.8%)	
SPONCS 4/4+	2 (2.0%)	10 (9.9%)	
SPONCS 5	2 (2.0%)	20 (19.8%)	
Pseudoexfoliation syndrome			0.065
Present	13 (12.9%)	34 (33.7%)	
Absent	7 (6.9%)	47 (46.5%)	
Diabetes			0.857
Present	6 (5.9%)	26 (25.7%)	
Absent	14 (13.9%)	55 (54.5%)	

Continuous variables were compared using independent *t*-tests, while categorical variables were analyzed with chi-square tests.

**Table 3:** Association between pseudoexfoliation syndrome status and patient demographics, cataract severity, diabetes, and glaucoma.

	PEXS (n = 47)	No PEXS (n = 54)	*p*-Value
Age, mean (SD)	77.7 ± 7.78	72.1 ± 9.33	**0.001**
Gender			0.634
Male	16 (15.8%)	16 (15.8%)	
Female	31 (30.78%)	38 (37.6%)	
Cataract severity			**0.011**
SPONCS 2/2+	9 (8.9%)	25 (24.8%)	
SPONCS 3/3+	22 (21.8%)	11 (10.9%)	
SPONCS 4/4+	5 (5.0%)	7 (6.9%)	
SPONCS 5	11 (10.9%)	11 (10.9%)	
Glaucoma			0.065
Present	13 (12.9%)	7 (6.9%)	
Absent	34 (33.7%)	47 (46.5%)	
Diabetes			0.417
Present	13 (12.9%)	19 (18.8%)	
Absent	34 (33.7%)	35 (34.7%)	

Continuous variables were compared using independent *t*-tests, while categorical variables were analyzed with chi-square tests. Bold *p*-values denote statistically significant differences (*p* < 0.05).

**Table 4:** Association between diabetes status and patient demographics, cataract severity, pseudoexfoliation syndrome, and glaucoma.

	Diabetes (n = 32)	No Diabetes (n = 69)	*p*-Value
Age, mean (SD)	75.0 ± 8.84	74.6 ± 9.11	0.823
Gender			0.057
Male	6 (5.9%)	26 (25.7%)	
Female	26 (25.7%)	43 (42.6%)	
Cataract severity			0.796
SPONCS 2/2+	9 (8.9%)	25 (24.8%)	
SPONCS 3/3+	11 (10.9%)	22 (21.8%)	
SPONCS 4/4+	5 (5.0%)	7 (6.9%)	
SPONCS 5	7 (6.9%)	15 (14.9%)	
Glaucoma			0.857
Present	6 (5.9%)	14 (13.9%)	
Absent	26 (25.7%)	55 (54.5%)	
PEXS			0.417
Present	13 (12.9%)	34 (33.7%)	
Absent	19 (18.8%)	35 (34.7%)	

Continuous variables were compared using independent *t*-tests, while categorical variables were analyzed with chi-square tests.

## Data Availability

The data used to support the findings of this study are available from the corresponding author upon reasonable request.

## Abbreviations

The following abbreviations are used in this manuscript:

KP	Kynurenine pathway
3-HK	3-hydroxykynurenine
KAT	Kynurenine aminotransferase
KYNA	Kynurenic acid
KMO	Kynurenine 3-monooxygenase
NMDA	*N*-methyl-D-aspartate
IDO	Indoleamine 2,3-dioxygenase
PEXS	Pseudoexfoliation syndrome
AGE	Advanced glycation end-product
ECM	Extracellular matrix
